# Sequence Data From a Travel-Associated Case of Microcephaly Highlight a Persisting Risk due to Zika Virus Circulation in Thailand

**DOI:** 10.1093/infdis/jiad322

**Published:** 2023-08-10

**Authors:** Solène Marquine, Guillaume André Durand, Gabriela Modenesi, Siham Khouadhria, Géraldine Piorkowski, Cyril Badaut, Thomas Canivez, Xavier De Lamballerie, Gilda Grard, Raphaëlle Klitting

**Affiliations:** Unité de Virologie, Institut de Recherche Biomédicale des Armées, Brétigny sur Orge, France; Unité des Virus Émergents, Aix-Marseille Univ–IRD 190–Inserm 1207, Marseille, France; National Reference Center for Arboviruses, National Institute of Health and Medical Research, and French Armed Forces Biomedical Research Institute, Marseille, France; Unité de Virologie, Institut de Recherche Biomédicale des Armées, Brétigny sur Orge, France; Unité des Virus Émergents, Aix-Marseille Univ–IRD 190–Inserm 1207, Marseille, France; National Reference Center for Arboviruses, National Institute of Health and Medical Research, and French Armed Forces Biomedical Research Institute, Marseille, France; Santé publique France, Saint-Denis, France; Agence Régionale de Santé, Saint-Denis, France; Unité des Virus Émergents, Aix-Marseille Univ–IRD 190–Inserm 1207, Marseille, France; Unité de Virologie, Institut de Recherche Biomédicale des Armées, Brétigny sur Orge, France; Unité des Virus Émergents, Aix-Marseille Univ–IRD 190–Inserm 1207, Marseille, France; Unité de Virologie, Institut de Recherche Biomédicale des Armées, Brétigny sur Orge, France; Unité des Virus Émergents, Aix-Marseille Univ–IRD 190–Inserm 1207, Marseille, France; National Reference Center for Arboviruses, National Institute of Health and Medical Research, and French Armed Forces Biomedical Research Institute, Marseille, France; Unité des Virus Émergents, Aix-Marseille Univ–IRD 190–Inserm 1207, Marseille, France; National Reference Center for Arboviruses, National Institute of Health and Medical Research, and French Armed Forces Biomedical Research Institute, Marseille, France; Unité de Virologie, Institut de Recherche Biomédicale des Armées, Brétigny sur Orge, France; Unité des Virus Émergents, Aix-Marseille Univ–IRD 190–Inserm 1207, Marseille, France; National Reference Center for Arboviruses, National Institute of Health and Medical Research, and French Armed Forces Biomedical Research Institute, Marseille, France; Unité des Virus Émergents, Aix-Marseille Univ–IRD 190–Inserm 1207, Marseille, France; National Reference Center for Arboviruses, National Institute of Health and Medical Research, and French Armed Forces Biomedical Research Institute, Marseille, France

**Keywords:** microcephaly, NGS, phylogenetic, Thailand, Zika virus

## Abstract

Zika virus has been circulating in Thailand since 2002 through continuous but likely low-level circulation. Here, we describe an infection in a pregnant woman who traveled to Thailand and South America during her pregnancy. By combining phylogenetic analysis with the patient's travel history and her pregnancy timeline, we confirmed that she likely got infected in Thailand at the end of 2021. This imported case of microcephaly highlights that Zika virus circulation in the country still constitutes a health risk, even in a year of lower incidence.

**Main points:**

Here we trace the origin of travel-acquired microcephaly to Thailand, providing additional evidence that pre-American lineages of Zika virus can harm the fetus and highlighting that Zika virus constitutes a health threat even in a year of lower incidence.

The first evidence of the presence of Zika virus (ZIKV; *Flavivirus* genus) in Asia dates back to 1966 in Malaysia [1]. It took almost 50 years before the virus was identified in the neighboring Thailand in 2013 [2]. Since then, surveillance, phylogenetic investigations [3], and infections detected in travelers (Supplementary Table 1) returning from the country suggest that the virus has persisted there through continuous but likely low-level circulation.

In 2022, the National Reference Center for Arboviruses (France) was asked by Santé publique France to confirm a probable infection with ZIKV in a 31-year-old patient whose fetus exhibited signs of microcephaly on ultrasound [4]. Pregnancy was terminated at 26 weeks due to the severity of the malformations, and tests performed on a fetal biopsy revealed high ZIKV RNA loads in the brain [4] (Figure 1).

**Figure 1.**
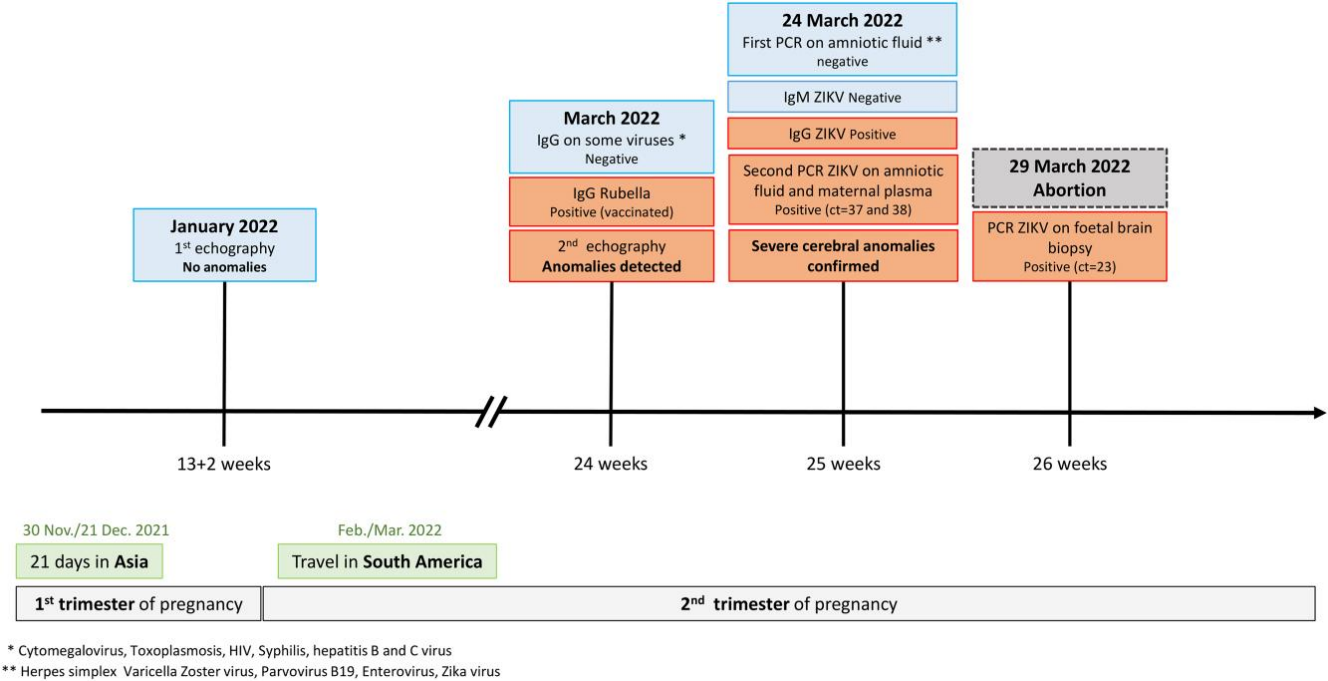
Timeline of the patient’s pregnancy, travels, and medical examinations. The pregnancy is shown by trimester (gray boxes) and week (ticks on the time axis). The trips made by the patient are shown by the green boxes, and the medical examinations that she underwent are detailed above the time arrow. The examinations and tests that yielded normal results are highlighted in blue, and those that revealed anomalies are highlighted in orange. ct, cycle threshold; IgG, immunoglobulin G; PCR, polymerase chain reaction; ZIKV, Zika virus.

Autochthonous cases are rare in France [5], and the patient had a history of travel to regions at risk of Zika in Asia and South America, suggesting that the infection was acquired abroad. The patient traveled to Thailand for 21 days during the first trimester of her pregnancy (November 30–December 21, 2021). She visited Bangkok, Chiang Mai, as well as the Ko Tao, Ko Samui, and Ko Pha Ngan islands (Supplementary Figure 1), but had no symptoms suggestive of ZIKV infection during and after her trip. She also traveled to Argentina and Uruguay during the second trimester (February–March 2022). As ZIKV-induced fetal damage is usually associated with infection during the first trimester, comparison of the history of travel with the pregnancy timeline suggested that the infection was contracted while the patient was in Thailand.

## METHODS

### Virus Isolation and Molecular Detection

RNA extraction was carried out with the QIAmp Viral RNA Mini Kit (Qiagen) according to the manufacturer's instructions. Five microliters of the eluted RNA were used to perform a quantitative reverse transcriptase–polymerase chain reaction (qRT-PCR) with 2 primers and a probe targeting the genome of ZIKV (Supplementary Table 2) as amplified through the SuperScript III Platinum One-Step qRT-PCR Kit (Thermo Fisher) on a LightCycler 2.0 Instrument (Roche Life Science, Mannheim).

### Extraction From Sample

RNA extraction was carried out with the QIAmp Viral RNA Mini Kit (Qiagen) according to the manufacturer's instructions.

### Virus Quantification From the Fetal Biopsy

Five microliters of the eluted RNA were used to perform a qRT-PCR with primers and probe targeting the ZIKV genome as amplified by the SuperScript III Platinum One-Step qRT-PCR Kit (Thermo Fisher) on a LightCycler 2.0 Instrument (Roche Life Science, Mannheim).

### Presequencing Amplification

We performed presequencing amplification of the viral genome with the RNA obtained from the fetal biopsy. Overlapping amplicons covering the entire virus genome were generated and amplified with the SuperScript IV One-Step RT-PCR System following the supplier's recommendations (Invitrogen). PCR mixes (final volume, 25 μL) contained 3 μL of RNA, 0.5 μL of each primer (Supplementary Table 2), and 12.5 μL of 2× Platinum SuperFi RT-PCR Master Mix. Amplification was performed with the following conditions: 10 minutes at 50 °C, 2 minutes at 98 °C, then 45 cycles of amplification (10 seconds at 98 °C, 10 seconds at 56 °C, and 2 minutes at 68 °C), and a final extension of 5 minutes at 68 °C. PCR products were verified by gel electrophoresis and pooled.

### Virus Sequencing

Sequencing was performed with S5 Ion torrent technology (Thermo Fisher Scientific). Briefly, samples were quantified with the Qubit dsDNA HS Assay Kit and Qubit 2.0 Fluorometer (Thermo Fisher Scientific). Libraries were built with the AB Library Builder System (Thermo Fisher Scientific) following the manufacturer's instructions, allowing for the barcoding the DNA for sample identification. Following real-time PCR quantification (Ion Library TaqMan Quantitation Kit; Thermo Fisher Scientific), equimolar pools of libraries were realized. The emulsion PCR of the pools and the loading on 520 chips were performed with the automated Ion Chef instrument (Thermo Fisher Scientific).

### Selection and Curation of Viral Sequences

All publicly available sequences for ZIKV were downloaded from the NCBI Nucleotide database (keyword “Zika virus”; accessed June 2022). We filtered the data by excluding laboratory strains (adapted, passaged multiple times, recombinant, obtained from antiviral or vaccine experiments) and sequences without a time stamp or collection location information. The remaining sequences were trimmed to their coding regions and aligned with MAFFT (version 7) and inspected manually with AliView (version 1.26). At this step, we discarded low-quality sequences (manual curation) and short sequences (open reading frame length <2500 nucleotides or >50% of undetermined nucleotides). Two types of alignments were generated: a *global* alignment, with all curated sequences regardless of their geographic origin (1123 sequences), and an *Asian* alignment, consisting of sequences originating exclusively in Asia or Oceania and with an open reading frame length >5000 nucleotides (206 sequences).

### Inferring ZIKV Phylogenies

We performed phylogenetic reconstructions using the global alignment under a GTR + F + R4 nucleotide substitution model implemented in iqtree2. Branch support was assessed with ultrafast bootstrap approximation (UFBoot2, 1000 replicates) and by optimizing each bootstrap tree with a hill-climbing nearest neighbor interchange search to limit branch support. To reconstruct a time-resolved phylogeny based on the Asian alignment, we first inferred a phylogenetic tree using iqtree2 as just described (model, UFBoot2, 1000 replicates) to examine the relationship between genetic divergence and time, and we found that we had a good temporal signal (correlation coefficient, 0.6802). We then inferred a time-resolved phylogeny using a bayesian approach with BEAST 1.10 coupled with the BEAGLE 3 library to improve computational performance. We modeled the nucleotide substitution process according to a GTR+Γ parameterization and the branch-specific evolutionary rates according to a relaxed molecular clock with an underlying log-normal distribution. We ran 4 independent Markov chain Monte Carlo chains for 50 million steps each and discarded the first 5 million steps as burn-in in each. We used Tracer version 1.7.1 to check for convergence, making sure that effective sample sizes were all >200. The .xml and .log files for this analysis are available at https://github.com/rklitting/Zika_case_report.

## RESULTS

### Phylogenetic Analyses Confirm an Infection of Thai Origin

**Figure 2.**
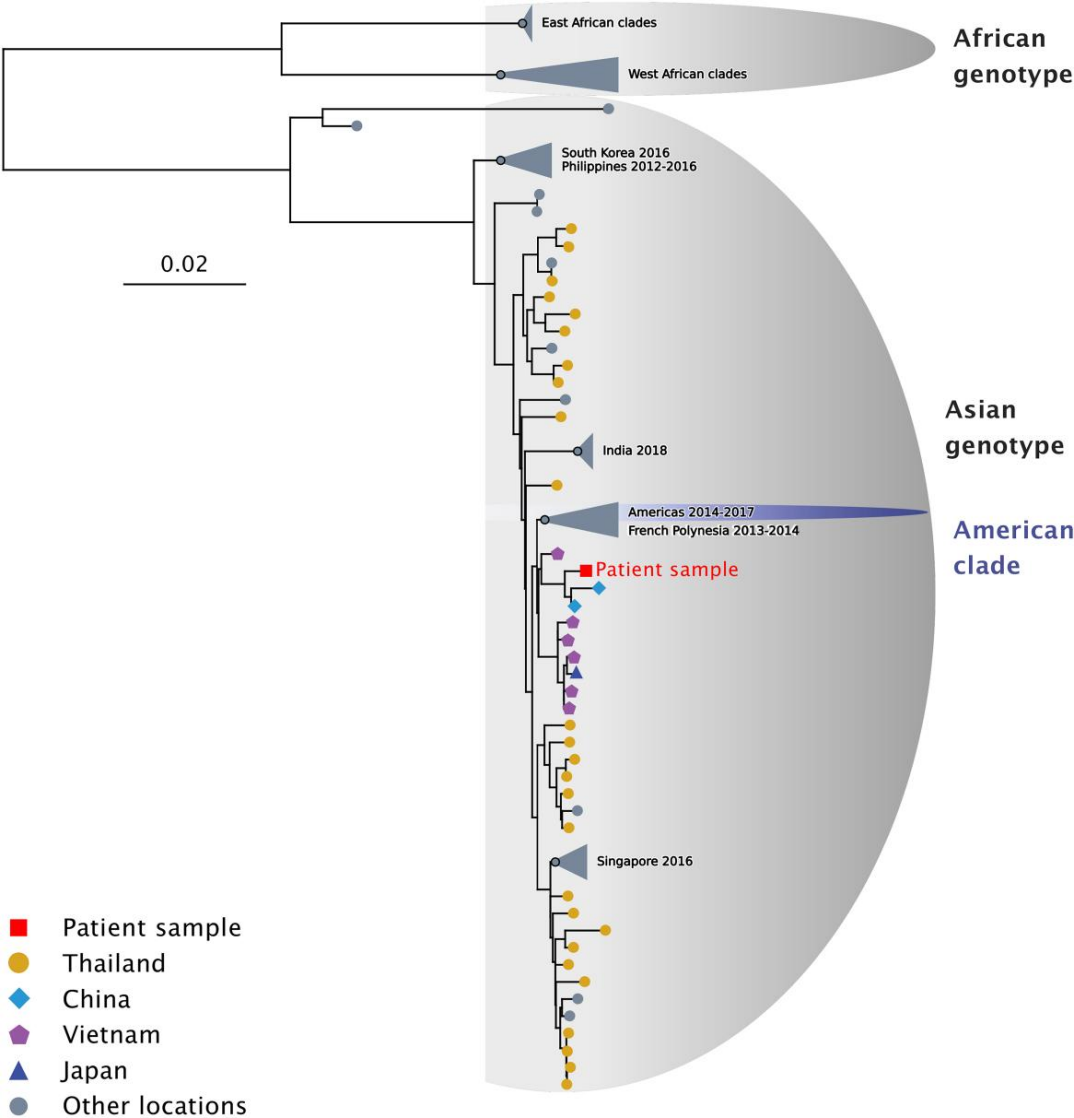
Situation of the patient sample among ZIKV sequences sampled globally. Phylogeny of ZIKV species obtained by a maximum likelihood approach under a GTR + F + R4 nucleotide substitution model implemented in iqtree2. Branch support was assessed with ultrafast bootstrap approximation (UFBoot2, 1000 replicates). The Asian and African genotypes are highlighted on the tree with gray semiellipses, and the American clade is highlighted by a purple semiellipse. The tip corresponding to the sequence obtained from the fetal biopsy is represented by a red square, while viral sequences sampled in Thailand, China, Vietnam, and Japan are represented by yellow circles, light blue diamonds, purple pentagons, and dark blue triangles, respectively. Collapsed clades and tips corresponding to sequences from other locations are shown in gray. ZIKV, Zika virus.

To discriminate between a potential Asian (Thailand) or American origin of the infection (Argentina, Uruguay), we sequenced the viral genome from the fetal biopsy (GenBank accession number OQ734448, BioProject PRJNA993116). To evaluate whether this genome was phylogenetically closer to virus sequences sampled in Asia or the Americas, we aligned it with a set of 1122 public sequences recapitulating the global genetic diversity within ZIKV species (available at https://github.com/rklitting/Zika_case_report) and inferred their phylogenetic relationships using a maximum likelihood approach. ZIKV species is divided into 2 major genotypes. The African genotype includes mainly strains from Africa, and the Asian genotype comprises strains from Southeast Asia, the Pacific Islands, and the Americas—the latter group in a separate phylogenetic clade (Figure 2). In the global phylogeny of ZIKV sequences, we found that the viral genome from the fetus belonged to the Asian genotype and grouped with sequences from the Asian region rather than those identified in the Pacific and the Americas, with good statistical support (bootstrap value > 95). These results indicated that the infection was most likely acquired in Thailand rather than Argentina or Uruguay.

To determine if the viral genome obtained from the patient belonged to lineages circulating in Thailand and to confirm the location of infection, we reconstructed a time-resolved phylogeny based on an alignment consisting exclusively of ZIKV sequences sampled in Asia. In the resulting phylogeny, the viral genome identified from the patient did not group with sequences previously identified in Thailand (Supplementary Figure 2) but clustered with 2 strains isolated in China in 2019 (Yunnan region, posterior probability > 0.9), including 1 corresponding to an import from Myanmar into China [6]. This small cluster was itself rooted by sequences from Vietnam sampled in 2016. These results, combined with the travel history and the pregnancy timeline, indicate that the patient was infected in Thailand in 2021 with a strain from a lineage circulating in Southeast Asia that had not been sampled in the country before.

## DISCUSSION

From 2016 to 2018, the Bureau of Epidemiology, Ministry of Public Health of Thailand, reported yearly ZIKV infections with an average incidence of 70 cases per month [7]. Since then, 142 cases were reported in 2019, 144 in 2020 [8], and 62 in 2021. While the COVID-19 pandemic may have affected the surveillance and epidemiology of Zika between 2020 and 2022, yearly case counts reported by the Thai Bureau of Epidemiology were higher in 2020 (n = 144) and 2022 (n = 191) than in 2021, suggesting that virus transmission was lower that specific year. This case report shows that the risk of health defects due to ZIKV infection remained in spite of the lower incidence, including for travelers. While most epidemiologic data, local case reports, and seroprevalence studies indicate that ZIKV is present in the continental parts of the country [7], little is known regarding virus circulation in the touristic islands of the Bandon Bay area. In this case report, the patient traveled to several islands in Bandon Bay but also visited locations in continental provinces with confirmed local cases of Zika (Bangkok, Chiang Mai, Surat Thani; Supplementary Figure 1); thus, it is more likely that the infection was acquired on the continent. However, repeated instances of infections in tourists with a history of travel to these islands suggest that the virus might also be present there [3, 9].

In this study, we found that the sequence retrieved from the patient's fetus appears to be most closely related to lineages circulating outside of Thailand (China, Myanmar, Vietnam) but remains distant even from these sequences. This important divergence can be explained by several factors: first, the sequence from the fetus belongs to a phylogenetic group that is itself characterized by long branches (Figure 2), suggesting that virus sequences from this group have been sampled sparsely, possibly due to locally limited surveillance and sequencing capacity; second, the virus sequence was determined on a fetal biopsy obtained around 4 months following the presumed time of infection. With several months of evolution under potentially weaker selective constraints in the fetus and the pregnant mother [10, 11], the virus genome may have diverged at a rate superior to what would have been observed under a “classical” arbovirus transmission cycle.

Using phylogenetic inference, we showed that the case of microcephaly described in this report was caused by a virus belonging to a pre-American lineage, bringing additional evidence that microcephaly is not exclusive to ZIKV American lineages [12]. It is possible that 1 or several sites in the ZIKV genome are involved in neuropathogenicity in the fetus. Previous studies have identified mutations that do exhibit properties in vitro and in vivo that suggest that they may participate in this process [13], although causal relationships are yet to be formally established [14]. To evaluate whether specific sites in the genome of the virus identified from the fetal biopsy could be linked with the development of microcephaly, we surveyed all the mutations potentially associated with ZIKV neuropathogenicity in the existing literature (Supplementary Table 3) and searched for them in the virus genome but found none. This result calls for further investigation into the determinants of ZIKV-induced microcephaly, particularly in pre-American lineages. Also, our finding highlights the fact that a virus genome that does not exhibit any genetic change suspected to be associated with neuropathogenicity can, however, be associated with severe microcephaly—all ZIKV strains should thus be considered a priori harmful for the fetus.

## CONCLUSIONS

This case report illustrates how molecular epidemiology can be effectively combined with patient history to pinpoint the source of an infection. Our results provide additional evidence that pre-American lineages of ZIKV can cause microcephaly, emphasizing the permanent risk posed by ZIKV circulation, even in years of lower incidence.

## Supplementary Data

Supplementary materials are available at *The Journal of Infectious Diseases* online. Consisting of data provided by the authors to benefit the reader, the posted materials are not copyedited and are the sole responsibility of the authors, so questions or comments should be addressed to the corresponding author.

## Supplementary Material

jiad322_Supplementary_Data

## Notes

***Author contributions***. S. M. and R. K. wrote the manuscript; R. K. performed the phylogenetic analyses; G. A. D., G. M., G. G., S. M., C. B., and S. K. investigated the case; S. M. and G. P. performed the sequencing; T. C. perform molecular analysis; X. D. L. supervised the findings of this work. All authors discussed the results and contributed to the final manuscript.

***Ethical statement***. Ethical review and approval were not required for the study of human participants in accordance with local legislation and institutional requirements. Written informed consent for participation was also not required for this study in accordance with national legislation and institutional requirements.

***Data availability***. https://github.com/rklitting/Zika_case_report.

***Financial support***. This work was supported by the French National Health Agency (Santé publique France) as well as the institutional bodies supervising the Unité des Virus Émergents (Institut national de la santé et de la recherche médicale, Institut de recherche pour le développement, Aix-Marseille Université, Service de Santé des Armées). Funding to pay the Open Access publication charges was provided by the Service de Santé des Armées.
