# Supplementary material for: Sequence Data From a Travel-Associated Case of Microcephaly Highlight a Persisting Risk due to Zika Virus Circulation in Thailand
Source: J Infect Dis. 2023 Aug 10;229(2):443–7. doi: 10.1093/infdis/jiad322 (PMC10873171; doi:10.1093/infdis/jiad322)
Supplement: jiad322_Supplementary_Data [file jiad322_supplementary_data.docx]

**Supplementary Table 1.** *Zika virus infections imported from Thailand.*

Sources for the year 2018 (1), 2019 (2)(3), 2016, 2019 and 2020 (3), 2019/2020 (4), 2022 (6)

| *Year* | Reported Imports from Thailand |
| --- | --- |
| *2018* | 4 |
| *2019* | 26 |
| *2020* | 2 |
| *2021* | 0 |
| *2022* | 5 |

1. C. Calba, « Arboviroses : données de surveillance pour anticiper la lutte / Arboviruses: Surveillance data to anticipate their control ».

2. S. Giron, « BILAN DE LA SURVEILLANCE DES ARBOVIROSES EN 2019 : TRANSITION VERS UNE SURVEILLANCE DES CAS CONFIRMÉS DE CHIKUNGUNYA, DENGUE ET D’INFECTION À VIRUS ZIKA EN FRANCE MÉTROPOLITAINE / REVIEW OF ARBOVIRUS SURVEILLANCE IN 2019: TRANSITION TO SURVEILLANCE FOR CONFIRMED CASES OF CHIKUNGUNYA, DENGUE AND ZIKA VIRUS IN METROPOLITAN FRANCE ».

3. « Promed Post », *ProMED-mail*. https://https://promedmail.org/promed-post/?id=6768177 (consulté le 14 mars 2023).

4. « Promed Post », *ProMED-mail*. https://https://promedmail.org/promed-post/?id=7156225 (consulté le 14 mars 2023). (5) « Travel-associated Zika virus disease cases in the EU/EEA and the UK ». https://www.ecdc.europa.eu/en/all-topics-z/zika-virus-infection/surveillance-and-disease-data/travel-associated-zika-virus (consulté le 14 mars 2023).

5. « Promed Post », *ProMED-mail*. https://https://promedmail.org/promed-post/?id=8704233 (consulté le 14 mars 2023).

**Supplementary Figure 1**. *Map of Thailand showing the location of provinces that have already reported Zika cases*

Provinces with cities visited by the patient are circled in red. Those with confirmed Zika cases are in red and those with suspected virus activity are in orange. Provinces in gray have not reported any cases. Until 2020, 42 provinces out of 76 are at least "at risk" [6-12]**.**

6. Fonseca K, Meatherall B, Zarra D, Drebot M, MacDonald J, Pabbaraju K, et al. First Case of Zika Virus Infection in a Returning Canadian Traveler. Am J Trop Med Hyg. 2014;91:1035–8.

7. Sirinam S, Chatchen S, Arunsodsai W, Guharat S, Limkittikul K. Seroprevalence of Zika Virus in Amphawa District, Thailand, after the 2016 Pandemic. Viruses. 2022;14:476.

8. Buathong R, Hermann L, Thaisomboonsuk B, Rutvisuttinunt W, Klungthong C, Chinnawirotpisan P, et al. Detection of Zika Virus Infection in Thailand, 2012–2014. Am J Trop Med Hyg. 2015;93:380–3.

9. Ruchusatsawat K, Wongjaroen P, Posanacharoen A, Rodriguez-Barraquer I, Sangkitporn S, Cummings DAT, et al. Long-term circulation of Zika virus in Thailand: an observational study. The Lancet Infectious Diseases. 2019;19:439–46.

10. Department of Disease Control weekly disease forecast No.116 Zika virus.

11. Sriburin P, Sittikul P, Kosoltanapiwat N, Sirinam S, Arunsodsai W, Sirivichayakul C, et al. Incidence of Zika Virus Infection from a Dengue Epidemiological Study of Children in Ratchaburi Province, Thailand. Viruses. 2021;13:1802.

12. Phumee A, Buathong R, Boonserm R, Intayot P, Aungsananta N, Jittmittraphap A, et al. Molecular Epidemiology and Genetic Diversity of Zika Virus from Field-Caught Mosquitoes in Various Regions of Thailand. Pathogens. 2019;8:30.

**Supplementary Figure 2. *Situation of the patient sample in the overall phylogeny of the ZIKV species****.* Phylogeny of ZIKV species obtained by bayesian inference based on 2000 posterior trees. Nodes with a posterior probability above 0.9 are shown by black circles. The tip corresponding to the sequence obtained from the fetal biopsy is shown by a full red circle and other tips are shown by circles colored according to the country of origin :


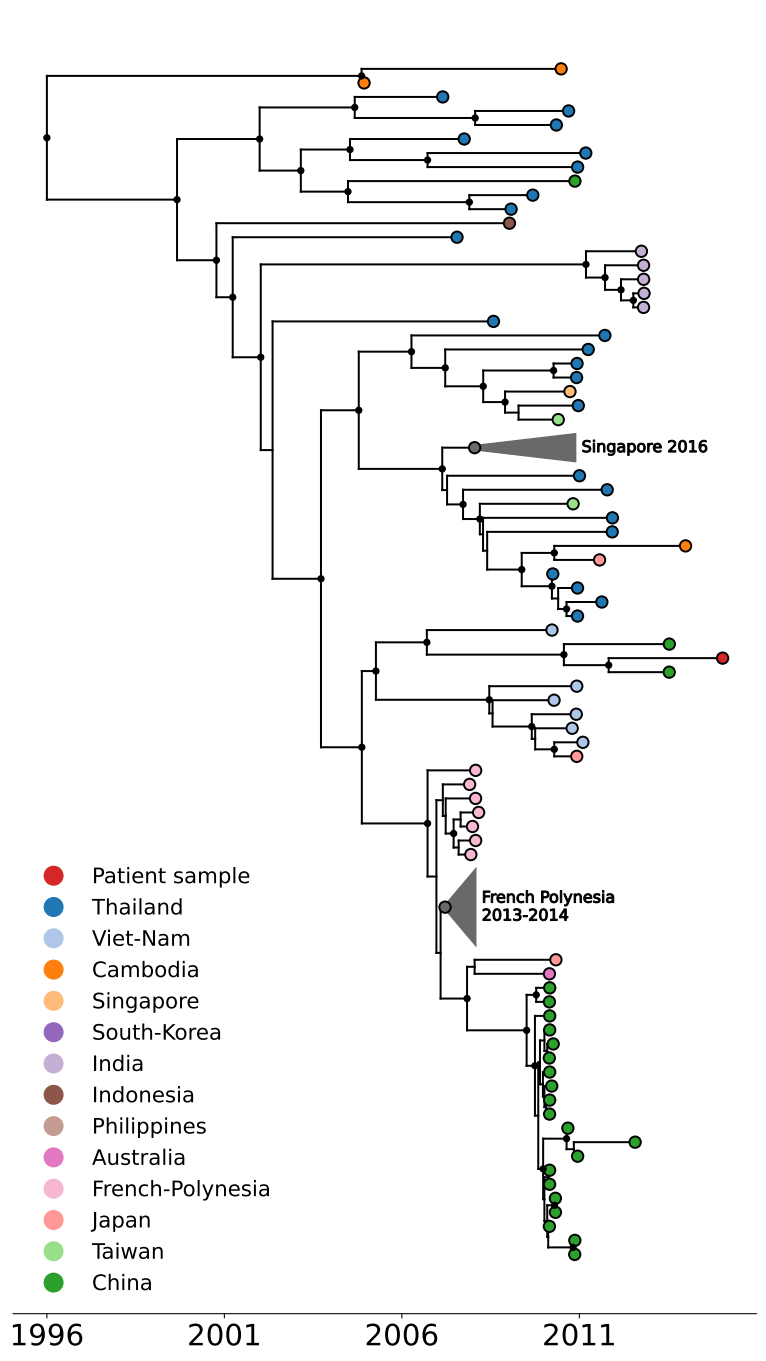


**Supplementary Table 2.** *RT-PCR and RT-qPCR primers*

| RT-PCR |  |
| --- | --- |
| Oligo name | Oligo sequence (5’ to 3’) |
| Forward 1 | ACT TGT IGA TCT GTG TGA GTC AG |
| Reverse 1 | TGT CCA ATT AGC TCT GAA GAT G |
| Forward 2 | AGT GCT TGT GAT TCT GCT CAT GGT |
| Reverse 2 | CAC AAA GTG GAA GTT GCS GCT GT |
| Forward 3 | ACG GCA GCT GGC ATC ATG AAG |
| Reverse 3 | TTC GGC GAT CTG TGC CTG G |
|  |  |
| ZIKA DUO Diag RT-qPCR |  |
| Oligo name | Oligo sequence (5’ to 3’) |
| ZIKA2-F | CTT GGA GTG CTT GTG ATT |
| ZIKA2-R | CTC CTC CAG TGT TCA TTT |
| ZIKA3-Probe_FAM | AAG AAG AGA ATG ACC ACA AAG ATC ATC |
| ZIKV-Bonn-NS1-S | CRA CYA CTG CAA GYG GAA GG |
| ZIKV-Bonn-NS1-R | GCC TTA TCT CCA TTC CAT ACC |
| ZIKV-Bonn-NS1-P_FAM | ATG GTG CTG YAG RGA RTG CAC AAT GC |

**Supplementary Table 3.** *Mutations potentially associated with ZIKV neuropathogenicity in the existing literature*

| **Mutation** | **Localisation** | **Effect** | **Presence** | **Reference** |
| --- | --- | --- | --- | --- |
| A123V * | PrM | Substantial effects on embryo development including increased rates of embryonic death and significant decrease in head diameter | No | Collette et al. 2020 |
| S139N | PrM | Increases microcephaly severity in mice fetuses ; increases ZIKV infectivity in both human and mouse neural progenitor cells ; emerged precisely when GBS and CZVS where first associated with ZIKV infection | No | Yuan et al. 2017; Petterson et al.2016 |
| A188V | NS1 | Increases NS1 Secretion and enhances mosquito infectivity phenotype, allows NS1 to inhibit the induction of interferon-β | No | Xia et al. 2018 ; Rossi et al. 2018 |
| T233A | NS1 | Disrupts the central hydrogen bonding network at the NS1 dimer surface and destabilizes NS1 dimer assembly in vitro | No | Wang et al. 2017 ; Rossi et al. 2018 |
| G894A | NS1 | Increases viremia (RNA copies) in mice and significantly increases embryo death ; causes a significant decrease in head diameter (potential contribution to Congenital Zika Syndrome after birth) | No | Collette et al. 2020 |
| A982V | NS1 | Increases NS1 secretion and enhances virus transmission from mice to mosquitoes in both mouse–mosquito and a mosquito-mouse–mosquito transmission models | Yes | Liu et al.2019 |
| M1404I | NS2B | Highly virulent, causes death in adult mice, abortions in pregnant females, and increases viral genome copies numbers in both brain tissue and blood of female mice | No | Collette et al. 2020 |
| M2074L | NS3 | Decreases embryo survival compared to epidemic strains | No | Collette et al. 2020 |
| H2086Y* | NS3 | Attenuated phenotype (in vitro and in vivo) as compared to epidemic strains | Yes | Collette et al. 2020 |
| M2634V | NS5 | Could have an impact on mosquito vector competence of epidemic ZIKV and increased ZIKV neurotropicity | No | Esser-Nobis et al. 2019 ; Mlakar et al. ; Liu et al. 2019 |
| * reversion |  |  |  |  |
